# Microbial community composition is related to soil biological and chemical properties and bacterial wilt outbreak

**DOI:** 10.1038/s41598-017-00472-6

**Published:** 2017-03-23

**Authors:** Rui Wang, Hongchun Zhang, Liguang Sun, Gaofu Qi, Shu Chen, Xiuyun Zhao

**Affiliations:** College of Life Science and Technology, Huazhong Agricultural University, Wuhan, 430070 China

## Abstract

Soil microbes play important roles in plant growth and health. Little is known about the differences of soil microbes between healthy and bacterial wilt infected soils with *Ralstonia solanacearum*. By Illumina-MiSeq sequencing of 16S rRNA and 18S rRNA gene amplicons, we found the soil microbial composition and diversity were distinct between healthy and bacterial wilt infected soils. Soil microbial community varied at different plant growth stages due to changes of root exudates composition and soil pH. Healthy soils exhibited higher microbial diversity than the bacterial wilt infected soils. More abundant beneficial microbes including *Bacillus*, *Agromyces*, *Micromonospora*, *Pseudonocardia*, *Acremonium*, *Lysobacter*, *Mesorhizobium*, *Microvirga*, *Bradyrhizobium*, *Acremonium* and *Chaetomium* were found in the healthy soils rather than the bacterial wilt infected soils. Compared to bacterial wilt infected soils, the activities of catalase, invertase and urease, as well as soil pH, available phosphorous and potassium content, were all significantly increased in the healthy soils. In a conclusion, the higher abundance of beneficial microbes are positively related the higher soil quality, including better plant growth, lower disease incidence, and higher nutrient contents, soil enzyme activities and soil pH.

## Introduction

Bacterial wilt caused by *Ralstonia solanacearum* is a soil-borne disease to infect tomato, hot pepper, egg plants, potato, tobacco, banana, *etc*., and causes serious economic losses worldwide^1^. Recently, bacterial wilt is more and more epidemic in China. Many efforts have been engaged in control of this disease^2–4^, but unfortunately it is still popular worldwide including China.

Various soil bio-chemical factors not only influence pathogen growth and survive in soils but also affect the nutrient availability for plant productivity. Among them, soil microorganisms are very important factors both for plants and pathogens, which play critical roles in regulating soil fertility, cycling of nutrients, promoting plant health and protecting plants from diseases such as bacterial wilt^5–7^. On the other hand, other soil bio-chemical factors such as soil pH also influence soil microbial community structure^8^. Healthy soils with balanced soil microbial community are beneficial for promoting plants growth and prevention of plant diseases^4, 9^. However, it is still unclear for the soil microbial community structure in the unhealthy soils, especially in the bacterial wilt infected soils.

The objective of this study is to evaluate the characteristics and differences between bacterial wilt infected soils and healthy soils, including soil biological and chemical properties, and soil microbial community. We hypothesized that: (1) soil properties are correlated with plant health, soil microbial community, and bacterial wilt; (2) soil microbial community is shifted in the bacterial wilt infected soils; (3) soil microbial community varies in the healthy and bacterial wilt infected soils at different plant growth stages. For this purpose, we examined the difference of microbial community of tobacco (used as a model plant in this study) planting soils via Illumina-MiSeq sequencing between unhealthy soils (bacterial wilt infected soils) and healthy soils, to elucidate the correlation of soil microbial community and soil health in the field.

## Results

### Soil biological and chemical properties

Soil biological and chemical properties were different between healthy and bacterial wilt infected soils. The activities of urease, invertase and catalase in healthy soils were significantly (*p* < 0.01) higher than the bacterial wilt infected soils from 30 to 90 d post-transplantation (Table 1), while the acid phosphatase activity was similar between healthy and bacterial wilt infected soils except for 30 d post-transplantation. At 30 d, the acid phosphatase activity was significantly (*p* < 0.05) lower in the healthy soils than the bacterial wilt infected soils (Fig. S1).

**Table 1:** Soil biological properties.

Time	Catalase (ml/g)		Invertase (mg/g)		Urease (mg/g)	
	Healthy soils	Bacterial wilt infected soils	Healthy soils	Bacterial wilt infected soils	Healthy soils	Bacterial wilt infected soils
**0 d**	2.36 ± 0.14 A	1.25 ± 0.14 B	41.80 ± 0.94 a	37.44 ± 1.34 b	0.19 ± 0.01 A	0.29 ± 0.01 B
**30 d**	2.19 ± 0.18 A	0.85 ± 0.14 B	53.40 ± 6.44 A	24.51 ± 7.68 B	0.23 ± 0.01 A	0.17 ± 0.02 B
**60 d**	1.78 ± 0.28 A	0.67 ± 0.07 B	35.64 ± 5.74 A	5.79 ± 1.01 B	0.35 ± 0.03 A	0.18 ± 0.01 B
**90 d**	2.06 ± 0.28 A	0.68 ± 0.09 B	76.70 ± 6.48 A	23.86 ± 2.48 B	0.31 ± 0.03 A	0.21 ± 0.01 B

All data are presented as the mean ± SE. Different capital letters and lowercase letters in the same line indicate very significant (*p* < 0.01) and significant (*p* < 0.05) difference between healthy and bacterial wilt infected soils, respectively.

The healthy soils contained less available N (AN) but more (*p* < 0.01) available P (AP) and K (AK) than the bacterial wilt infected soils from 30 d to 90 d post-transplantation (Table 2). The AK and AP content had the similar variable trends both in healthy and bacterial wilt infected soils, which reached the highest level at 30 d and 60 d, respectively, then both decreased thereafter until the end. The soil pH in healthy soils was significantly (*p* < 0.05) higher than the bacterial wilt infected soils from 30 d to 90 d post-transplantation (Table 2). However, there was no significant difference of soil organic matter (SOM) content between healthy and bacterial wilt infected soils from 30 d to 90 d (Fig. S1).

**Table 2:** Soil chemical properties.

Time	AN (mg/Kg)		pH		AP (mg/Kg)		AK (mg/Kg)	
	Healthy soils	Bacterial wilt infected soils	Healthy soils	Bacterial wilt infected soils	Healthy soils	Bacterial wilt infected soils	Healthy soils	Bacterial wilt infected soils
**0 d**	146.57 ± 2.58 a	152.62 ± 5.28 a	6.2 ± 0.1 a	5.8 ± 0.1 a	33.61 ± 1.16 A	26.04 ± 0.23 B	275.00 ± 0 a	265.0 ± 0 a
**30 d**	155.46 ± 2.35 a	159.59 ± 2.32 a	5.28 ± 0.09 a	4.94 ± 0.12 b	80.43 ± 7.88 A	38.99 ± 3.89 B	1547.04 ± 151.32 A	794.36 ± 61.59 B
**60 d**	158.50 ± 4.07 A	173.23 ± 2.38 B	5.08 ± 0.27 a	4.52 ± 0.12 b	86.86 ± 4.03 A	45.11 ± 4.85 B	1399.19 ± 90.91 A	848.12 ± 34.92 B
**90 d**	153.21 ± 6.62 a	168.78 ± 3.97 b	5.48 ± 0.26 A	4.67 ± 0.04 B	75.37 ± 4.04 A	32.14 ± 4.37 B	1163.98 ± 114.64 A	639.79 ± 64.81 B

All data are presented as the mean ± SE. AK: available potassium content; AN: available nitrogen content, AP: available phosphorous content. Different capital letters and lowercase letters in the same line indicate very significant (*p* < 0.01) and significant (*p* < 0.05) difference between healthy and bacterial wilt infected soils, respectively.

### Plant growth and disease incidence

Tobaccos grew better in the healthy soils than the bacterial wilt infected soils. The height and stem circumference of tobaccos were significantly (*p* < 0.01) higher in the healthy soils than the bacterial wilt infected soils (Fig. S2). In the infected soils, severe bacterial wilt occurred, with significantly higher disease incidence than the healthy soils from 30 d to 90 d after transplantation. The disease index of bacterial wilt infected soils was 16.54 and 93.82 at 60 and 90 d post-transplantation, respectively, while it was 0 at all time points for the healthy soils (Table 3).

**Table 3:** Disease incidence of tobacco bacterial wilt.

Time	Disease index		Disease incidence (%)	
	Healthy soils	Bacterial wilt infected soils	Healthy soils	Bacterial wilt infected soils
30 d	0	0.27 ± 0.02**	0	2.4 ± 0.46**
60 d	0	16.54 ± 0.77**	0	42.22 ± 0.91**
90 d	0	93.82 ± 0.52**	0	100 ± 0**

**Significant at *p* < 0.01 level.

### Soil bacterial diversity

Totally, 207426 effective sequence reads were obtained from all soil samples after filtering out low-quality reads and chimera sequences. The operational taxonomic units (OTUs) number, Chao 1, and Shannon index were used to evaluate and compare the diversity and richness of bacterial communities among different soil samples (Table 4). The number of OTUs in all soil samples ranged from 3538 to 6611, and the 16S rRNA gene diversity was higher in the healthy soils than the bacterial wilt infected soils. From 0 to 90 d post-transplantation, the number of OTUs and Shannon index were both higher in the healthy soils than the bacterial wilt infected soils, indicating the diversity of soil bacteria was higher in the healthy soils than the bacterial wilt infected soils. Analysis by Chao1, a higher richness of bacteria was also found in the healthy soils.

**Table 4:** Number of OTUs and alpha diversity of bacteria.

Sample	Times	Number of OTUs	Chao 1	Shannon index
Healthy soils	0 d	6611	8026	7.96
	30 d	6202	6993	7.70
	60 d	5291	6197	7.38
	90 d	5915	7426	7.57
Bacterial wilt infected soils	0 d	6513	7394	7.40
	30 d	4244	4937	6.72
	60 d	3538	4822	6.85
	90 d	5366	7346	7.34

We further analyzed the temporal trends of OTUs number and Shannon index in the soils, and found they firstly decreased from 0 d to 60 d then increased at 90 d after transplantation both in healthy and bacterial wilt infected soils (Table 4). At 60 d, OTUs number decreased to 54.3% of the initial (0 d) in the bacterial wilt infected soil, possibly due to low soil pH at this time (Table 2). The overlapping bacterial OTUs between healthy and bacterial wilt infected soils were 11.6% (1145/9874) at 60 d post-transplantation (Fig. S3). More specific OTUs were found in the healthy soils (5052) than the bacterial wilt infected soils (3677). This result showed the soil bacterial diversity varied dramatically in the healthy soils when compared to the bacterial wilt infected soils.

### Bacterial community composition

Bacteria were identified as 26 phyla in all soil samples. *Proteobacteria* was the most abundant phylum (27%), followed by *Actinobacteria* (14%), *Acidobacteria* (14%), *Chloroflexi* (8%) and *Firmicutes* (6%) (Fig. S4). Except for *Acidobacteria*, *Chlamydiae*, *Chloroflexi* and *Spirochaetes*, other phyla were more abundant in the healthy soils than the bacterial wilt infected soils (Fig. S4).

**Figure 1:**
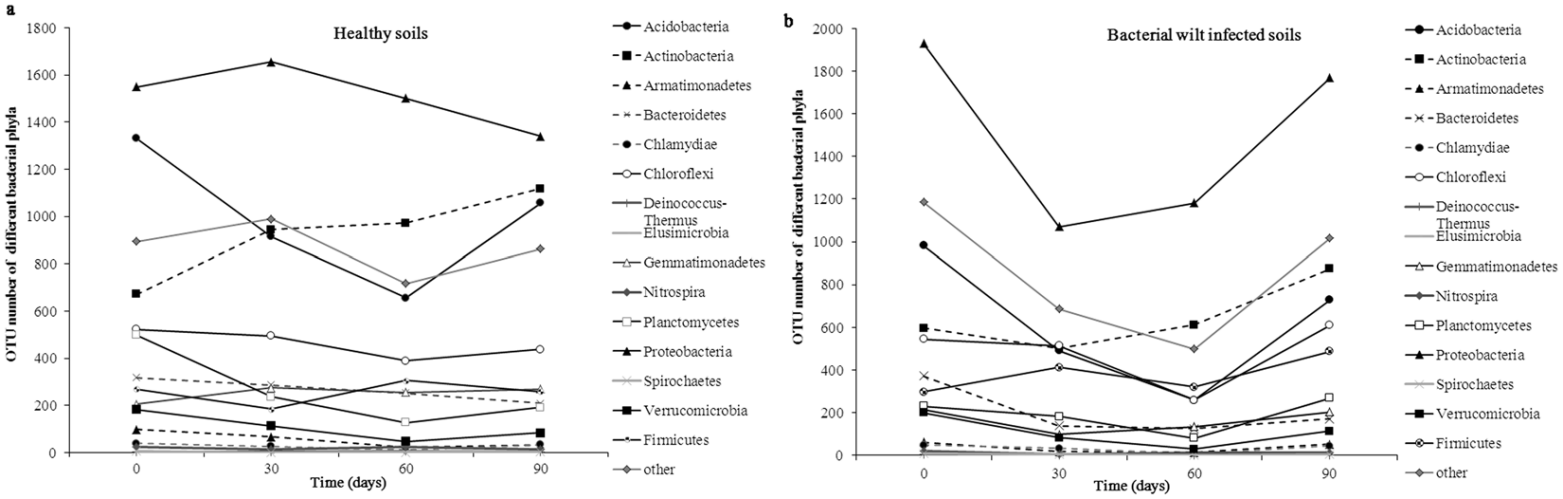
Temporal trends of different bacterial phyla in healthy soils (**a**) and bacterial wilt infected soils (**b**) during the different tobacco growth stages.

The abundance of bacterial phyla changed during different tobacco growth stages both in healthy soils and bacterial wilt infected soils (Fig. 1). For example, the abundance of *Proteobacteria* increased in the healthy soils at 30 d, while obviously decreased in the bacterial wilt infected soils at 30 d and 60 d. The abundance of *Actinobacteria* increased continuously from 0 d to 90 d in the healthy soils, while decreased at 30 d and then increased at 60 and 90 d in the bacterial wilt infected soils.

### Different bacterial communities in the healthy and bacterial wilt infected soils

**Figure 2:**
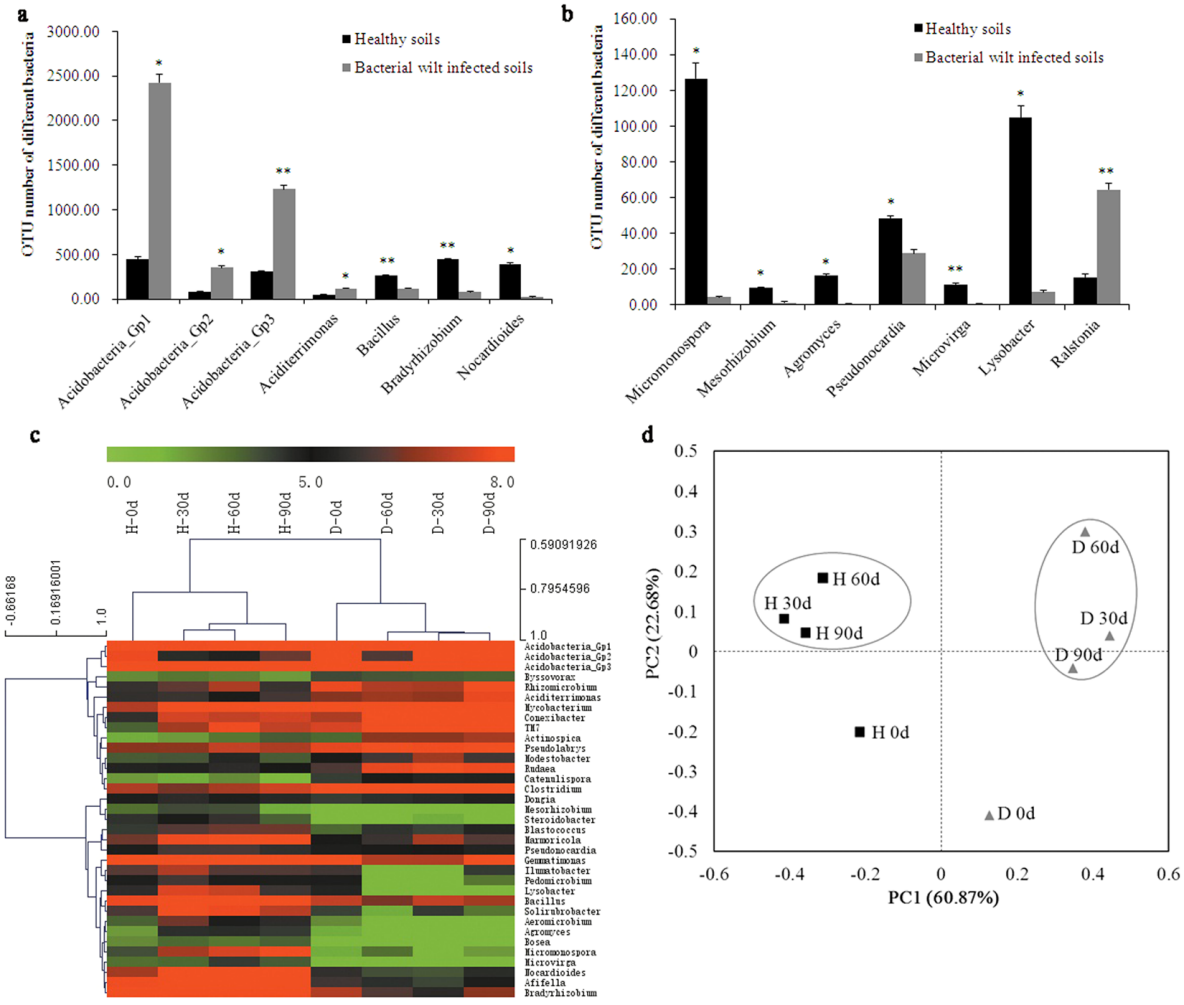
Comparison of abundance of different soil bacterial genera and bacterial community between healthy and bacterial wilt infected soils. (**a**) and (**b**) Abundances of different soil bacterial genera were compared between healthy and bacterial wilt infected soils; (**c**) Relative abundance and hierarchical cluster analysis of bacterial genera; (**d**) PCoA analysis of soil bacterial community. H: healthy soils; D: bacterial wilt infected soils. Bars with asterisk (*) and double asterisks (**) indicate significant (*p* < 0.05) and very significant (*p* < 0.01) difference between healthy and bacterial wilt infected soils, respectively.

The bacterial difference between healthy and bacterial wilt infected soils was analyzed at the genus level. Abundances of 36 bacterial genera were significantly (*p* < 0.05) different between healthy and bacterial wilt infected soils (Fig. 2, Fig. S4), that was indicated by Heatmaps and hierarchical clustering analysis (Fig. 2). 19 bacterial genera were more abundant in the healthy soils, including *Aeromicrobium*, *Agromyces*, *Bacillus*, *Blastococcus*, *Gemmatimonas*, *Micromonospora*, *Nocardioides*, *Pseudonocardia*, *Solirubrobacter*, *Bradyrhizobium*, *etc*., while the rest 17 genera including *Ralstonia*, *Aciditerrimonas*, *Actinospica*, *Byssovorax*, *Catenulispora*, *Conexibacter*, *Dongia*, *Acidobacteria Gp1*-*Gp3*, *Modestobacter*, *Mycobacterium*, *Pseudolabrys*, *Rhizomicrobium*, *Rudaea* and *Clostridium* were more abundant in the bacterial wilt infected soils. Interestingly, the beneficial microorganisms (*e*.*g*. *Bacillus*, *Bradyrhizobium*, *Nocardioides* and *Micromonospora*) were more abundant in the healthy soils than the bacterial wilt infected soils.

Principal co-ordinates analysis (PCoA) showed the bacterial community were different between healthy soils and bacterial wilt infected soils at different time points. PC1 and PC2 explained 83.55% of the total bacterial community (Fig. 2). Healthy soils (H 30 d, H 60 d, and H 90 d) and bacterial wilt infected soils (D 30 d, D 60 d and D 90 d) were respectively clustered together and separated from each other at PC1 axis. In the healthy soils, H 0 d was well separated from other three time points (H 30 d, H 60 d, and H 90 d) at PC2 axis. D 0 d was also separated from other three time points (D 30 d, D 60 d and D 90 d) in the bacterial wilt infected soils, indicating the soil bacterial community changed obviously after transplanting.

### Shifts of soil fungal diversity and community structure in bacterial wilt infected soils

**Figure 3:**
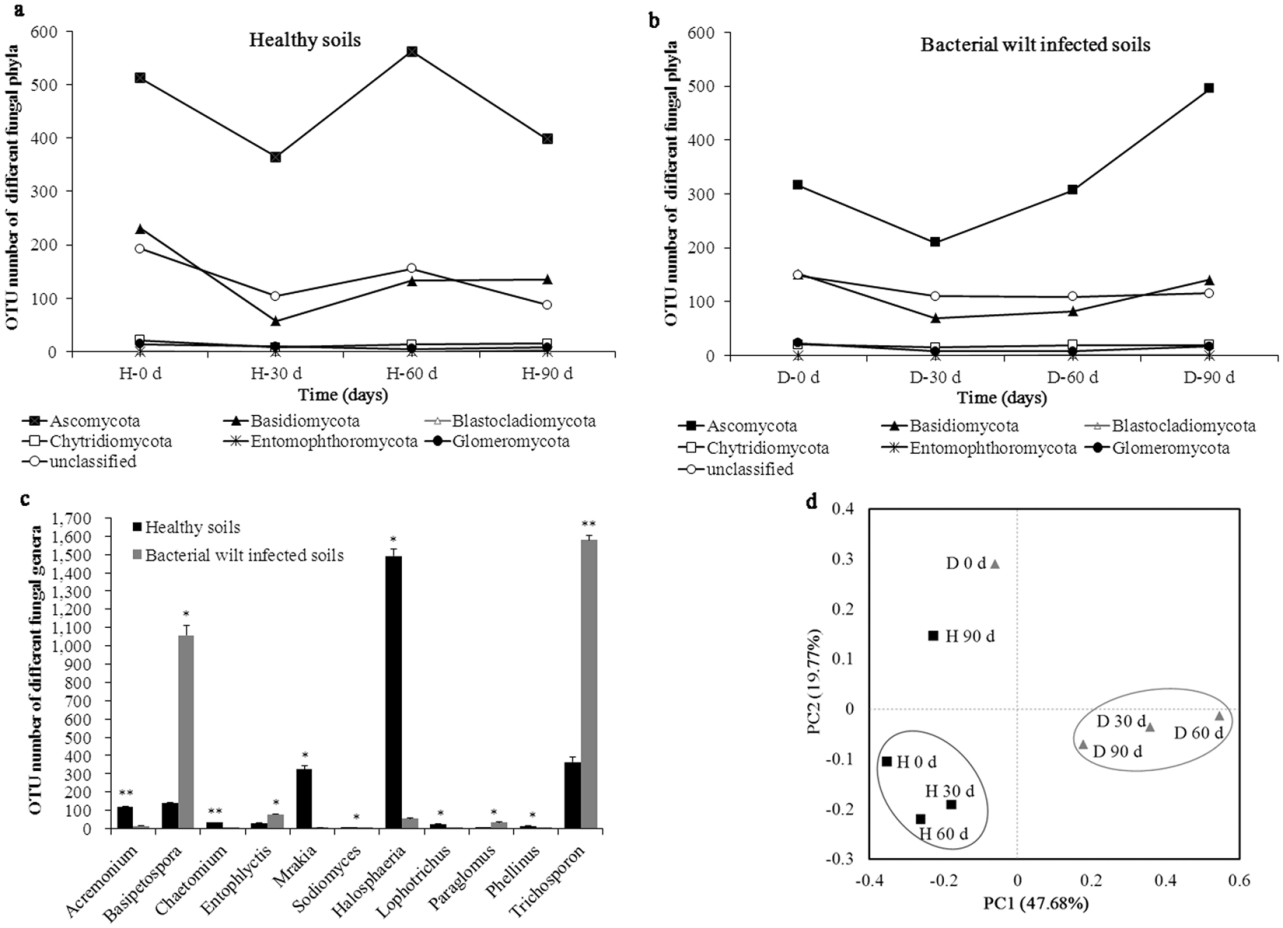
Soil fungal diversity and community structure. Temporal trends of different fungal phyla in healthy soils (**a**) and bacterial wilt infected soils (**b**) during different tobacco growth stages; (**c**) Abundance of different fungal genera were compared between healthy and bacterial wilt infected soils. Bars with asterisk (*) and double asterisks (**) indicate significant (*p* < 0.05) and very significant (*p* < 0.01) difference between healthy and bacterial wilt infected soils, respectively; (**d**) PCoA analysis of soil fungal community. H: healthy soils; D: bacterial wilt infected soils.

All soil samples consist of 14235 fungal OTUs and 292463 reads. Sequences from fungi matched 6 main known phyla, including *Ascomycota* (58.35%) followed by *Basidiomycota* (18.47%) (Fig. S5). The temporal trends of fungal phyla in healthy soils were obviously different from the bacterial wilt infected soils (Fig. 3). OTUs number of different fungal phyla firstly decreased then increased in the bacterial wilt infected soils after transplantation; however, it varied irregularly in the healthy soils. For example, the abundance of *Ascomycota* decreased at 30 d, increased at 60 and decreased again at 90 d in the healthy soils, while it decreased at 30 d then continuously increased from 60 to 90 d in the bacterial wilt infected soils.

The fungal OTUs number ranged from 414 to 999 in different soil samples, much less than the soil bacterial abundance (Table 5). The healthy soil samples had higher OTUs number and Shannon index during all stages of tobacco growth when compared to the bacterial wilt infected soils. The average Shannon index was 4.3 in the healthy soils, much higher than the bacterial wilt infected soils of 3.4. Analysis by Chao1, it was found the healthy soils also had a higher fungal richness than the bacterial wilt infected soils (Table 5). Thereby, the healthy soils have a higher fungal diversity and richness than the bacterial wilt infected soils.

**Table 5:** Number of OTUs and alpha diversity of fungi.

Sample	Times	Number of OTUs	Chao 1	Shannon index
Healthy soils	0 d	999	3209	4.4
	30 d	553	1986	4.3
	60 d	879	3149	4.4
	90 d	659	1672	4.1
Bacterial wilt infected soils	0 d	671	1786	3.7
	30 d	414	1065	3.3
	60 d	537	1655	2.7
	90 d	601	1665	3.5

The percentage of overlapping fungal OTUs between healthy and bacterial wilt infected soils was only 5.28% (241/4563) at 60 d post-transplantation (Fig. S3), indicating these two different soil types have a great number of specific fungi species (2908 for healthy soils, and 1414 for bacterial wilt infected soils). This result showed the soil fungal types varied dramatically in the healthy soils when compared to the bacterial wilt infected soils.

The abundances of 11 fungal genera were significantly (*p* < 0.05) different between healthy and bacterial wilt infected soils (Fig. 3, Fig. S5). In the healthy soils, the abundances of *Acremonium*, *Chaetomium*, *Sodiomyces*, *Halosphaeria*, *Lophotrichus*, *Mrakia* and *Phellinus* were significantly (*p* < 0.05) higher, while the abundances of *Basipetospora*, *Trichosporon*, *Paraglomus* and *Entophlyctis* were significantly (*p* < 0.05) lower than the bacterial wilt infected soils. Further analysis found some beneficial fungi (*e*.*g*. *Chaetomium* and *Acremonium*) were more abundant in the healthy soils than the bacterial wilt infected soils, which are favorable for improving soil quality and inhibiting soil pathogens^10–12^.

PCoA analysis showed the soil fungal communities of healthy soils were different from the bacterial wilt infected soils. PC1 and PC2 explained 67.45% of the fungal community. Figure 3 clearly showed the healthy soils (H 0 d, H 30 d, and H 60 d) were well separated from the bacterial wilt infected soils (D 30 d, D 60 d, and D 90 d) at PC1 axis. Healthy soils (H 0 d, H 30 d, and H 60 d) and bacterial wilt infected soils (D 30 d, D 60 d, and D 90 d) were clustered together, respectively. H 90 d and D 0 d were separated alone. The results indicated the soil fungal community structure of bacterial wilt infected soils was dramatically shifted when compared with the healthy soils. The plant growth stages also obviously impacted on the dynamic and diversity of soil fungal community.

### Relationships between microbial community structure and environmental variables

**Figure 4:**
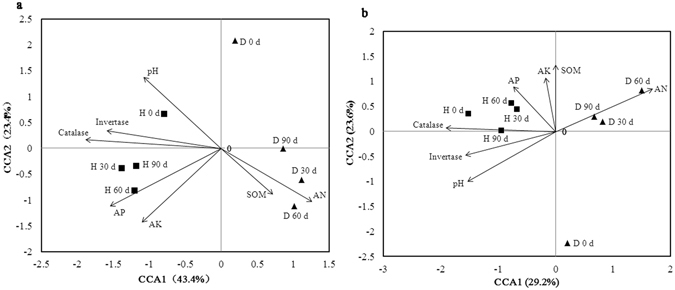
Canonical correspondence analysis of the relationship between microbial community structure and soil properties. (**a**) soil bacterial community; (**b**) soil fungal community. The soil properties are indicated with arrows, including soil pH, invertase, catalase, soil organic matter (SOM), available potassium (AK), nitrogen (AN), and phosphorous (AP) content. H: healthy soils; D: Bacterial wilt infected soils. The percentage of variation is explained by each axis.

The relationships between microbial community structure and soil bio-chemical properties were analyzed with canonical correspondence analysis (CCA). Seven parameters including pH, invertase, catalase, soil organic matter (SOM), AK, AN and AP were selected for CCA based on the significant test, and the results showed 66.8% of community variation could be explained by these variables (Fig. 4). The healthy soils and bacterial wilt infected soils were grouped separately, and we found the first canonical axis (CCA1) was positively correlated with AN and soil organic matter, and the second canonical axis (CCA2) was positively correlated with pH, invertase and catalase, but negatively correlated with AP and AK. As important variables (represented by longer arrows), AP, AN, AK, catalase, invertase and pH play major roles in the shaping of soil bacterial community structure. The healthy soils (H 30 d, H 60 d and H 90 d) were positively correlated with AP and AK, while the bacterial wilt infected soils were positively correlated with AN but negatively correlated with pH, AP, AK, invertase and catalase.

For fungi (Fig. 4), 52.8% of community variation can be explained by the variables. The healthy and bacterial wilt infected soils were grouped separately along CCA1. The first canonical axis was positively correlated with AN, and the second axis was positively correlated with AP, AK, SOM and catalase. As important variables, AN, pH, invertase and catalase played major roles in the shaping of soil fungal community structure. The healthy soils were positively correlated with AP and catalase, while the bacterial wilt infected soils (D 30 d, D 60 d and D 90 d) positively correlated with AN, and negatively correlated with pH, invertase and catalase.

## Discussion

We hypothesized that soil properties are correlated with plant health, soil microbial community, and bacterial wilt. Here, we found soil bio-chemical properties were relevant to soil microbial community. Compared to bacterial wilt infected soils, the activities of soil oxidoreductase (catalase), and enzymes involved in C (invertase) and N (urease) cycling were significantly enhanced in the healthy soils, which are deduced to release more soil nutrients for plants and soil microorganisms. P additions can cause an increase of bacterial diversity, with increased *Actinobacteria* and *Alphaproteobacteria*, and decreased *Acidobacteria* in soils^13, 14^. Here, lower abundance of *Acidobacteria*, and higher abundance of *Proteobacteria*, *Actinobacteria*, *Firmicutes* and *Bacteroidetes* were found in the healthy soils with higher AP content. In addition, nitrogen is one of the important factors for plant growth. The optimal nitrogen application rate (168–210 kg/hm^−2^) can improve tobacco growth, while the excessive application of nitrogen fertilizers has negative effect on plant growth by decreasing soil pH^15–17^. We found the AN content was positively correlated with the microbial community of bacterial wilt infected soils, indicating the AN content may be an indicator for soil microbial community and plant health.

It was found that the bacterial wilt infected soils were acidic with a lower bacterial and fungal diversity than the healthy soils. As reported, soil pH strongly influences the composition of soil microbial community. The soils with near - neutral pH have a higher bacterial diversity than the acidic ones, and the abundances of many bacterial phyla are strongly correlated with soil pH^18, 19^. Soil acidity is linked to the decrease of available carbon for soil microbes^20^; thereby, the soil pH may act as an environmental filter (by stressing microbial cells) for selecting specific microbial groups and regulating soil microbial community composition^21^. Here, the healthy soils with high pH value had a higher abundance of *Actinobacteria* and *Bacteroidetes* than the bacterial wilt infected soils, consistently with the reports^19^. Conversely, the bacterial wilt infected soils had a higher abundance of *Acidobacteria* Gp1, Gp2, Gp3, and *Aciditerrimonas* (Fig. 2), which are potential indicators for acidic and diseased soils^9, 21, 22^. Notably, the low soil pH is positively correlated with *R*. *solanacearum* epidemic^23^, which can stress tobaccos making them more susceptible to infection of *R*. *solanacearum*.

We also hypothesized that the soil microbial community is shifted in the bacterial wilt infected soils, which was proven in this study. The soil bacterial and fungal communities of healthy soils were very different from the bacterial wilt infected soils. We also found the healthy soils had higher microbial diversity, as reported, the soil microbial diversity is positively correlated with the plant resistance to pathogens^24–26^. Healthy soils had higher abundances of beneficial microbes (*e*.*g*. *Bacillus*, *Agromyces*, *Micromonospora*, *Pseudonocardia*, *Acremonium*, *Lysobacter*, *Bradyrhizobium*, *Mesorhizobium*, *Microvirga*, *Acremonium*, and *Chaetomium*), which can improve soil nutrients, promote plant growth and control soil-borne diseases^27^. For example, *Bacillus* is generally effective for suppressing bacterial wilt caused by *R*. *solanacearum* ^28–30^. *Agromyces*, *Micromonospora* and *Pseudonocardia* play important roles in degradation of xylan through production of xylanase^31–33^. *Micromonospora*, *Acremonium* and *Lysobacter* can produce antimicrobial compounds to protect plants from pathogens infection^34–36^. *Bradyrhizobium* is reported to suppress fungal pathogens and root-knot nematodes^37^. *Mesorhizobium*, *Microvirga* and *Bradyrhizobium* are beneficial for plants growth by nitrogen-fixation^38^. Both *Acremonium* and *Chaetomium* are potential for bio-control of plant disease via production of lytic enzymes and antimicrobial metabolites^10–12^. Thereby, we infer these beneficial bacteria and fungi are positively correlated with soil quality and plant health. Whereas, *Ralstonia* is more abundant in the bacterial wilt infected soils, which can cause serious bacterial wilt in the tobacco field.

Lastly, we hypothesized that the soil microbial community varies in healthy and bacterial wilt infected soils at different plant growth stages. Here, we found the soil microbial communities were distinct at different tobacco growth stages. The composition of root exudates differs at different plant growth stages, with strong impact on soil microbial communities^39, 40^. For example, phenol and phenolic acids have been identified in the tobacco root exudates^41, 42^, which may greatly impact on microbial biomass and diversity in soils^43, 44^. Here, the bacterial OTUs number increased at 90 d post-transplantation, this may be explained by the increased tobacco root exudates for promoting soil bacterial growth at this stage. It is noted that some bacterial (*e*.*g*. *Proteobacteria*, *Actinobacteria*) and fungal (*e*.*g*. *Ascomycota*, *Basidiomycota*) abundances obviously changed with different trends at different tobacco growth stages in healthy and bacterial wilt infected soils. *Proteobacteria* play key roles in the soil carbon, sulfur and nitrogen cycles, including purple nonsulfur bacteria (*Rhodospirillum* and *Rhodopseudomonas*)^45^, free-living aerobic nitrogen fixers (*Azotobacter*, *Azomonas*, *Azospirillum* and *Beyerinckia*)^46^, and nitrifying bacteria (*Nitrobacter*, *Nitrococcus* and *Nitrospira*)^47^. The abundance of *Proteobacteria* in healthy soils increased in 30 d because fertilizers are adequate at this stage, that may be beneficial for promoting the C, N, S cycles in soils. However, the abundance of *Proteobacteria* in healthy soils decreased at 60 and 90 d, because most of fertilizers were exhausted and the composition of root exudates also changed at these stages. On the other hand, the abundance of *Proteobacteria* greatly decreased in the bacterial wilt infected soils at 30 d possibly due to poor soil environments such as soil acidification in the tobacco field. Most of *Actinobacteria* can produce antibiotics inhibiting plant pathogens and controlling plant diseases^48^. The abundance of *Actinobacteria* increased continuously from 0 d to 90 d in the healthy soils, and always were higher than the bacterial wilt infected soils at all time points.

In addition, the abundance of *Ascomycota* was higher in the healthy soils than the bacterial wilt infected soils from 0 d to 60 d, which can enhance soil C cycle and plant nutrient uptake^49, 50^. The abundance of *Basidiomycota* decreased at 30 d and then increased at 60 d and 90 d in the healthy soils, while it decreased both at 30 and 60 d in the bacterial wilt infected soils. Both *Ascomycetes* and *Basidiomycota* are important decomposers in carbon cycle, which can secrete digestive enzymes to break down organic substances (*e*.*g*. cellulose, lignocellulose, lignin in plant litters) into smaller molecules^49, 50^. Thereby, the increase of *Basidiomycota* abundance is favorable for degradation of plant litters and promotion of C cycle in soils^49, 50^.

In conclusions, we found the soil microbial composition and diversity were distinct between healthy and bacterial wilt infected soils. The soil microbial community varied at different plant growth stages possibly due to the changed root exudates and soil pH. Healthy soils exhibited a higher microbial diversity and abundance of beneficial microbes such as *Bacillus*, *Agromyces*, *Micromonospora*, *Pseudonocardia*, *Acremonium*, *Lysobacter*, *Mesorhizobium*, *Microvirga*, *Bradyrhizobium*, *Acremonium* and *Chaetomium*, but a lower abundance of pathogens such as *R*. *solanacearum*. High abundances of beneficial microbes are positively related with the high soil quality, which is indicated by better plant growth, lower disease incidence, and higher soil pH, nutrient and enzyme activities.

## Materials and Methods

### Soil sampling

The study site was located in Enshi county (29.97°N, 109.38°W), Hubei province, central China. The fields are tobacco planting soils with subtropical humid climate (an annual rainfall of 1400–1500 mm and annual average temperature of 16 °C). Tobacco has been cultivated continuously for more than 15 years. Tobacco in certain fields exhibited severe bacterial wilt infected by *R*. *solanacearum*, whereas tobacco in other fields grew well without bacterial wilt. The soil samples were collected separately from three healthy fields in which the tobacco grew well without bacterial wilt (healthy soils), and three bacterial wilt infected fields where the tobaccos exhibited severe bacterial wilt. Bulk soil samples were obtained from the healthy and bacterial wilt infected soils at four time points (0 d - transplanting stage, 30 d - rosette stage, 60 d - fast growing stage, and 90 d - maturation stage post-transplantation) (Fig. S6). For each field (667 m^2^), a composite sample was collected from a mixture of 20 random soil cores (5 cm diameter, 15 cm depth). Each soil sample was partitioned into two subsamples, one for DNA extraction and another for analysis of bio-chemical properties after air-dry.

### Analysis of soil bio-chemical properties

Urease, catalase, acid phosphatase and invertase were respectively measured according to Guan^51^. After being suspended with water (soil: water = 1: 2.5, w/v), the soil pH was measured using a pH meter (Mettler-Toledo FE20; Switzerland). Soil available N, P, K, and organic matter (SOM) were respectively measured by the methods described by Bao^52^. All tests were performed in triplicates.

### Tobacco growth and disease incidence of bacterial wilt

Tobacco stem circumference and height, as well as disease incidence and disease index of bacterial wilt, were recorded at 90 d post-transplantation, respectively. Disease incidence was calculated by the percentage of diseased tobaccos in each field. Disease index was evaluated using a disease score method: 0 = no symptom, 1 = below one half of tobacco leaves wilted, 3 = one half to two-thirds of tobacco leaves wilted, 5 = above two-thirds of tobacco leaves wilted, 7 = all leaves wilted, and 9 = stems collapsed or tobaccos died^53^. Disease index was calculated using the formula:

$${\rm{Disease}}\,{\rm{index}}=[\sum (r\times N)/(n\times R)]\times 100$$

*r* is the disease severity, *N* is the number of infected tobaccos with a rating of *r*, *n* is the total number of tobaccos tested, and *R* is the value of the highest disease severity in each field.

### Soil DNA extraction

0.4 g soils were used to extract soil microbial genomic DNA using soil DNA extraction kits (FastDNA Spin Kit, MP Biomedicals, USA) according to the manufacturer’s instructions. DNA concentration was determined using a NanoDrop ND-1000 spectrophotometer (NanoDrop Technologies Inc., Wilmington, DE, USA).

### Microbial rRNA gene amplification and Illumina sequencing

The extracted soil genomic DNA was used as template to amplify 16S rRNA and 18S rRNA genes, respectively. The V3 - V4 regions of 16S rRNA gene were amplified using primers 338F (5′-ACTCCTACGGGAGGCAGCA-3′) and 806R (5′-GGACTACHVGGGTWTCTAAT-3′)^54^, and the V5 - V7 regions of 18S rRNA gene were amplified using primers 0817F (5′-TTAGCATGGAATAATRRAATAGGA-3′) and 1196R (5′-TCTGGACCTGGTGAGTTTCC-3′)^55^. Amplicons were sequenced on Illumina-MiSeq platform (Illumina Inc., USA) at SHBIO Technology (Shanghai, China). The sequence quality was statistically analyzed by CASAVA1.8. The raw sequence data was preliminarily filtrated using the FASTX Toolkit 0.0.13 software package, removing the low mass base at the tail of the sequence (Q value less than 20), and finally removing the sequences with lengths less than 35 bp. The length of the valid reads was approximately 250 bp.

### Statistical analysis

The operational taxonomic units (OTUs) at 97% similarity were used to perform rarefaction analysis and calculate the richness and diversity index. Sample normalization was conducted by rarefaction. Chao and Shannon indices were calculated as measures of microbial richness and diversity. The taxonomic differences between groups were compared by least-significant-difference (LSD) test with Holm-Bonferroni adjustment. *P* < 0.05 was considered with statistical significance. Principal co-ordinates analysis (PCoA) was performed based on OTU data to calculate the difference of microbial communities between healthy and bacterial wilt infected soils. We also performed hierarchical clustering analysis by Multi-experiment Viewer 4.9.0 based on microorganisms with significant difference between health and bacterial wilt infected soils. Heatmaps were generated using custom *R* scripts. Canonical correspondence analysis (CCA) using vegan package in *R* version 3.2.5 is performed to analyze the relationships between microbial community structure and environmental variables. Seven environmental factors including pH, catalase, invertase, AP, AN, SOM and AK were chosen to perform CCA analysis.

## Electronic supplementary material

Supplement Figures
