# Supplementary material for: Microbial community composition is related to soil biological and chemical properties and bacterial wilt outbreak
Source: Sci Rep. 2017 Mar 23;7:343. doi: 10.1038/s41598-017-00472-6 (PMC5428506; doi:10.1038/s41598-017-00472-6)

Microbial community composition is related to soil biological and chemical properties and bacterial wilt outbreak

Running title: Microbial communities shifted in bacterial wilt infected soils

Rui Wang[§], Hongchun Zhang[§], Liguang Sun, Gaofu Qi, Shu Chen, Xiuyun Zhao[*]

College of Life Science and Technology, Huazhong Agricultural University, Wuhan 430070, China;

* Correspondence author, E-mail: xiuyunzh@mail.hzau.edu.cn; Tel: +86-15387157410.

[§] These authors contributed equally to this work.

Figure S1 Analysis of acid phosphatase activity (a) and soil organic matter content (b) in the healthy and bacterial wilt infected soils.

a

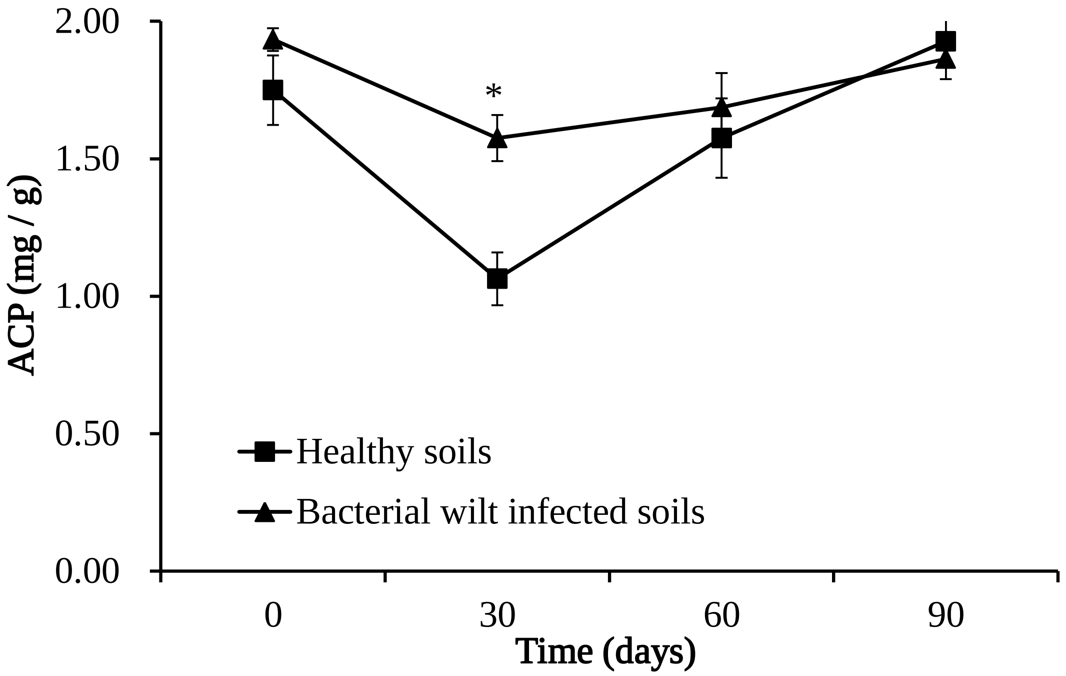

b

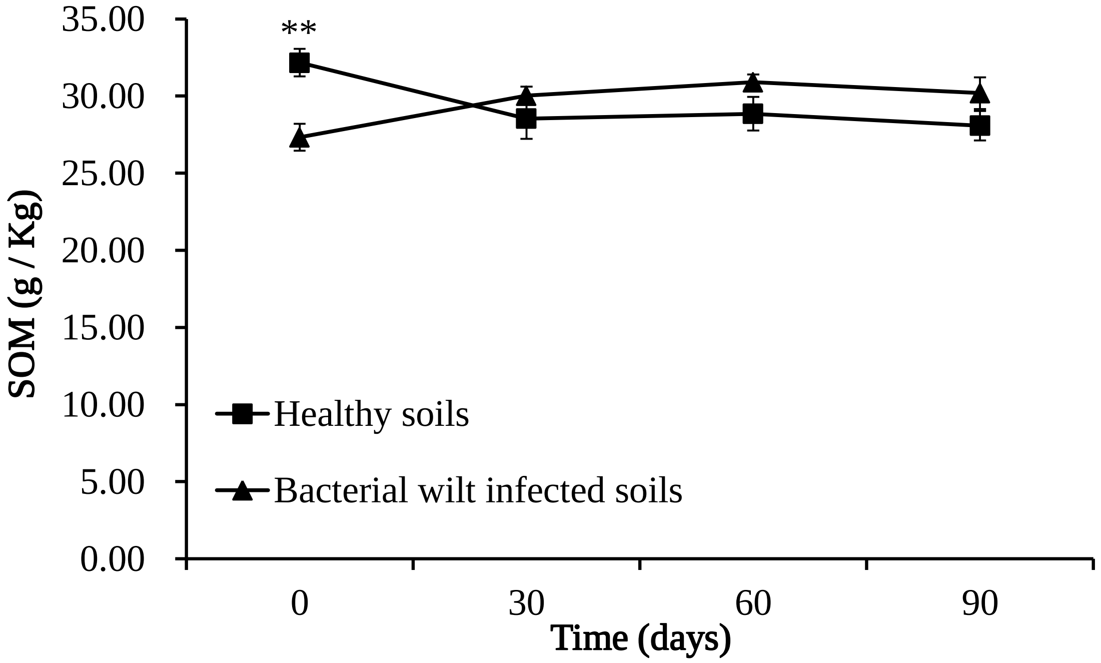

Figure S2 Tobacco growth characters in healthy and bacterial wilt infected soils. Plant height (a) and stem circumference (b) of tobacco in healthy soils (H) was compared to that in bacterial wilt infected soils (D). Bars with different capital letters indicate significant ($p < 0.01$) difference between healthy and bacterial wilt infected soils.

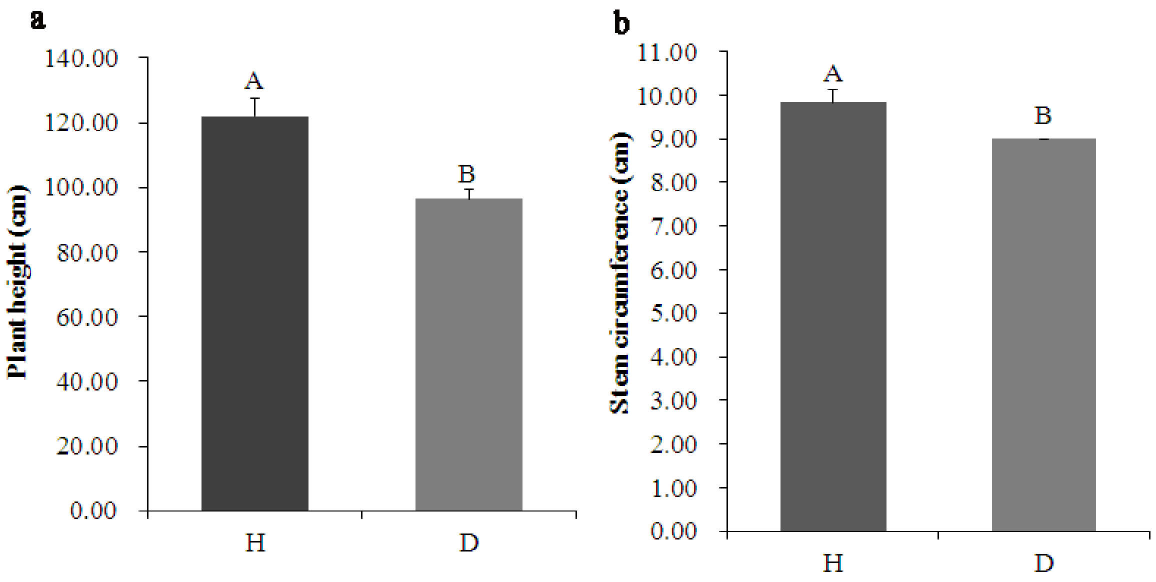

Figure S3 Venn diagram showing the unique numbers of bacterial OTUs (a) and fungal OTUs (b) detected in healthy and bacterial wilt infected soils. H: healthy soils. D: bacterial wilt infected soils.

a

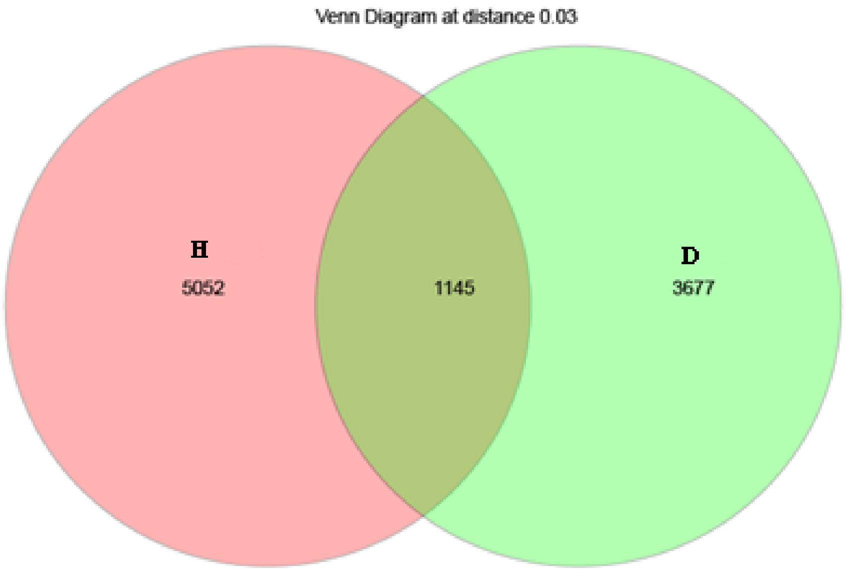

b

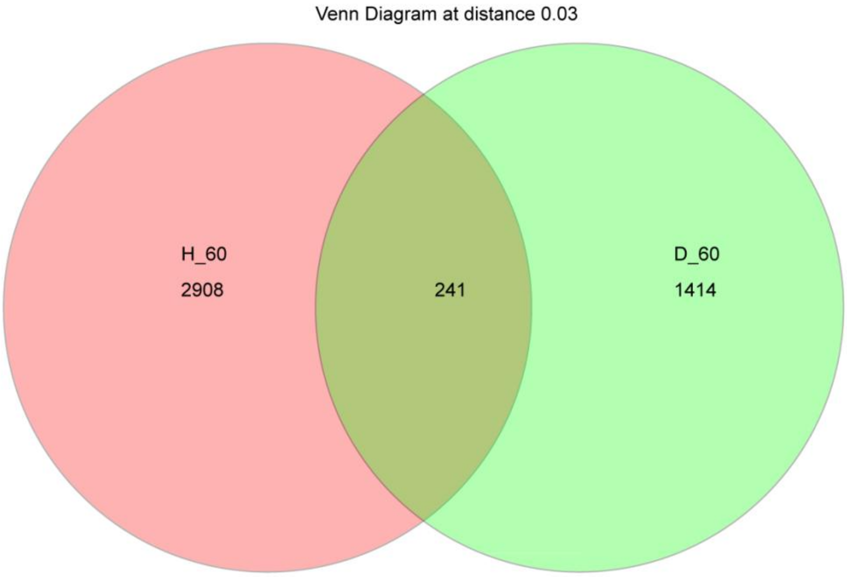

Figure S4 Abundance of different soil bacterial phyla and genera. (a) Relative abundance of different bacterial phyla in all soil samples; (b) Relative abundances of the dominant bacterial phylum in healthy soils (H) and bacterial wilt infected soil (D) samples; (c) Abundances of different soil bacterial genera were compared between healthy and bacterial wilt infected soils.

a

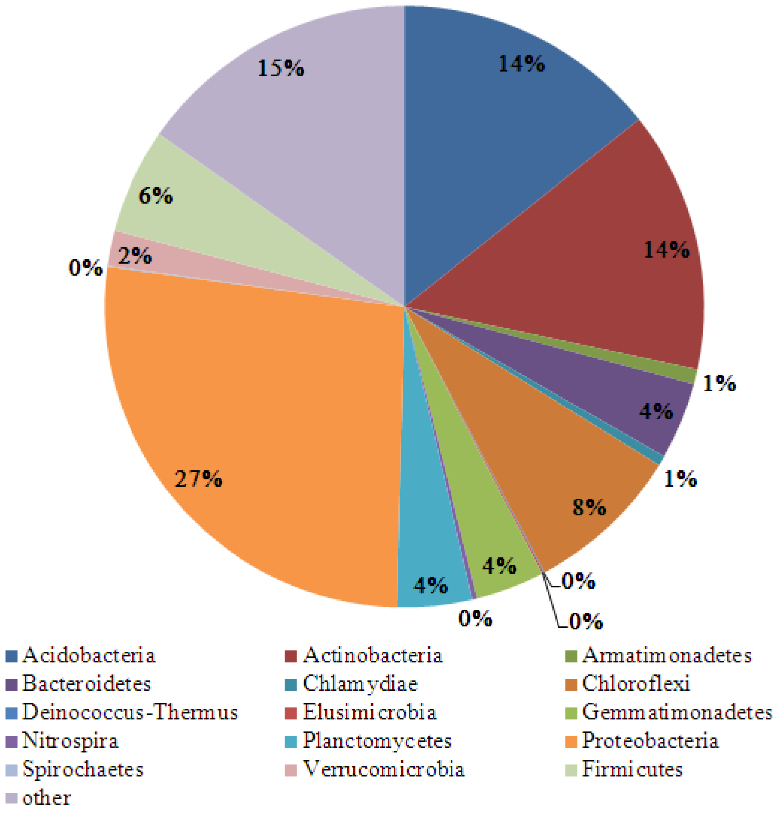

b

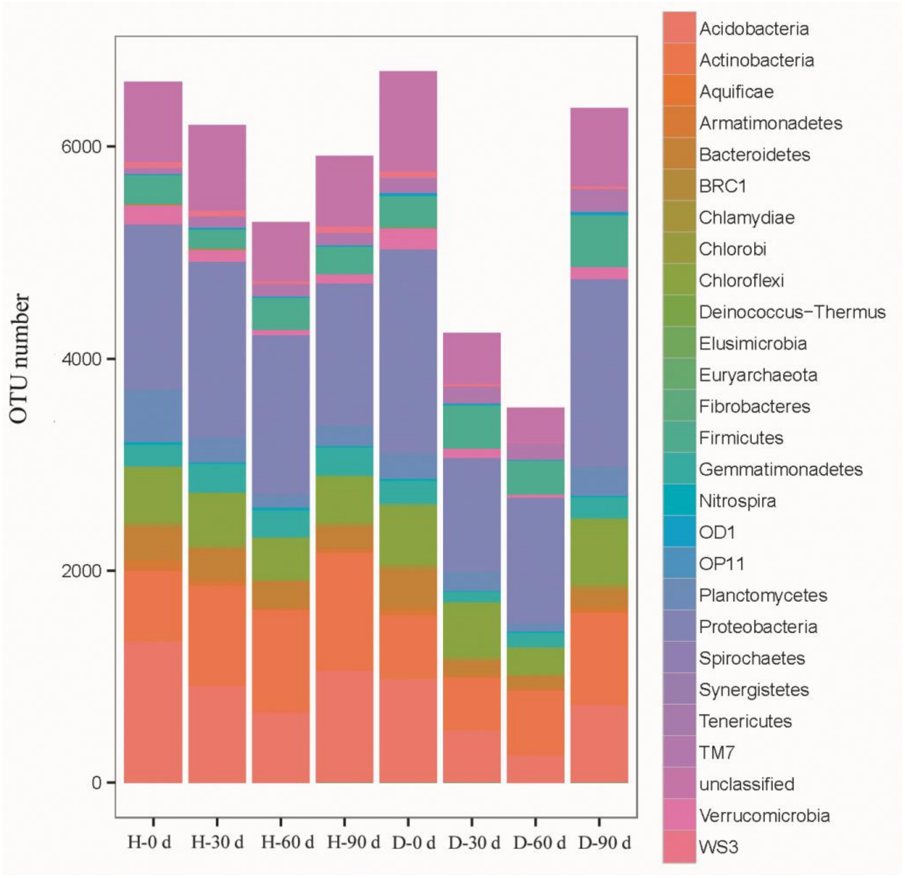

OTU number
6000
4000
2000
0
H-0 d
H-30 d
H-60 d
H-90 d
D-0 d
D-30 d
D-60 d
D-90 d
Acidobacteria
Actinobacteria
Aquificae
Armatimonadetes
Bacteroidetes
BRC1
Chlamydiae
Chlorobi
Chloroflexi
Deinococcus-Thermus
Elusimicrobia
Euryarchaeota
Fibrobacteres
Firmicutes
Gemmatimonadetes
Nitrospira
OD1
OP11
Planctomycetes
Proteobacteria
Spirochaetes
Synergistetes
Tenericutes
TM7
unclassified
Verrucomicrobia
WS3

c

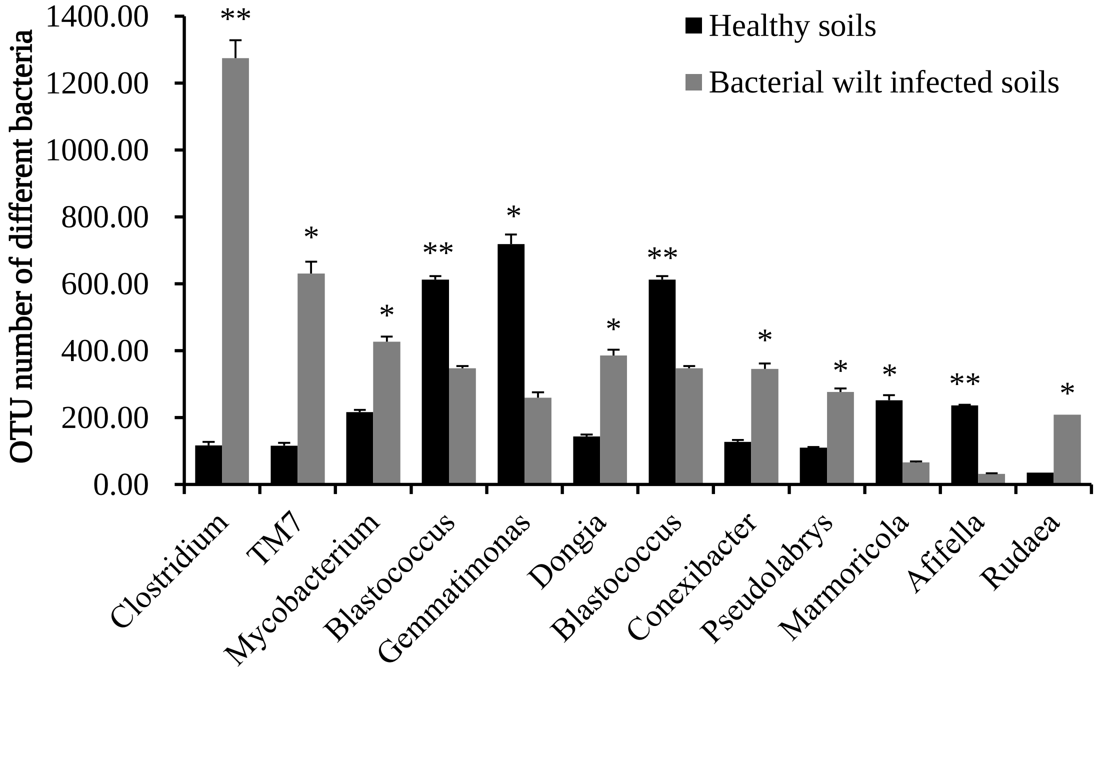

OTU number of different bacteria
1400.00
1200.00
1000.00
800.00
600.00
400.00
200.00
0.00
Healthy soils
Bacterial wilt infected soils
**
*
*
**
*
*
**
*
*
*
**
*
Clostridium
TM7
Mycobacterium
Blastococcus
Gemmatimonas
Dongia
Blastococcus
Conexibacter
Pseudolabrys
Marmoricola
Afifella
Rudaea

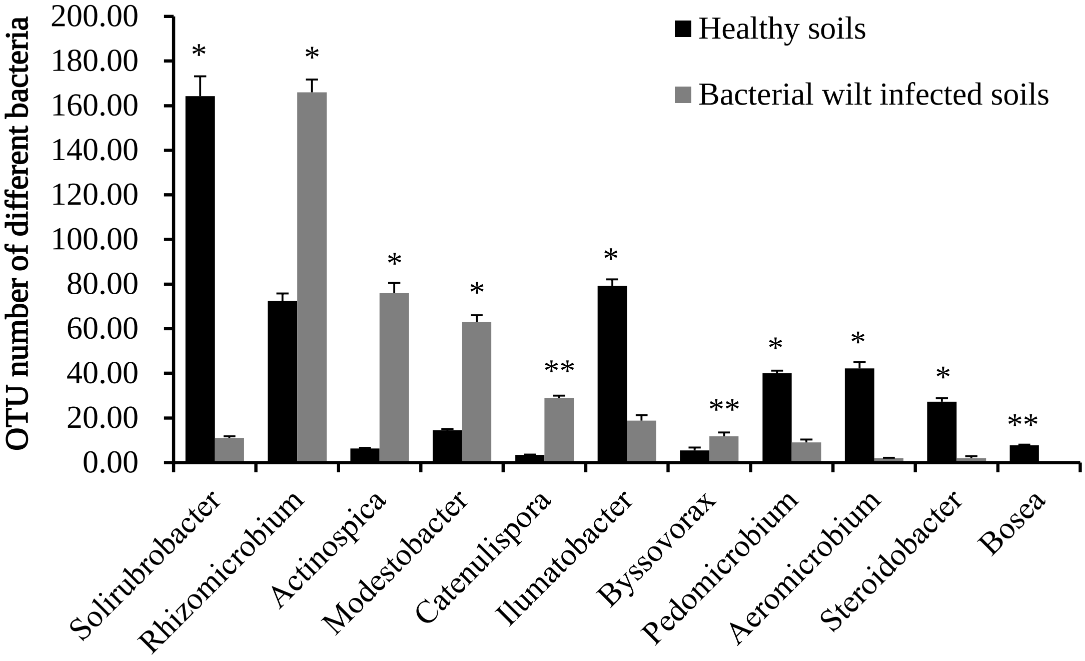

Figure S5 Abundance of different soil fungal phyla and genera. (a) Relative abundance of different fungal phyla in all soil samples; (b) Relative abundance and hierarchical cluster analysis of 11 fungal genera. H: healthy soils. D: bacterial wilt infected soils.

a

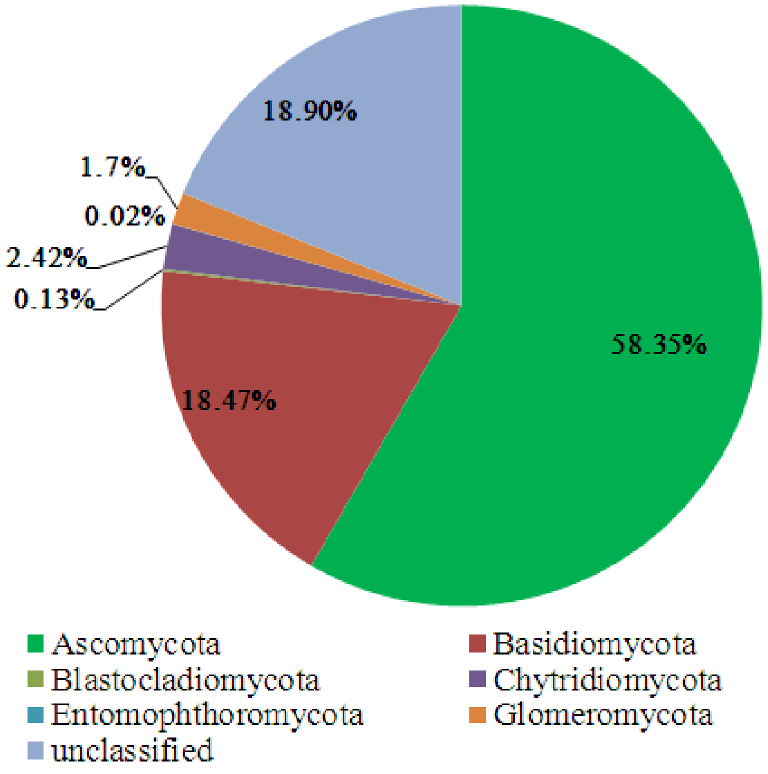

b

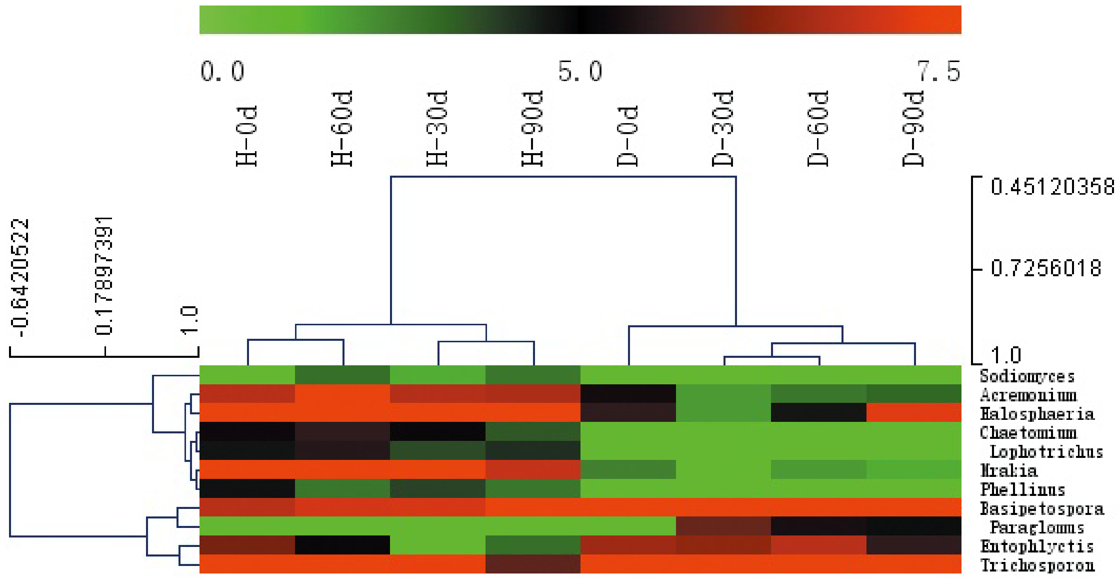

Figure S6 Soil samples collection.

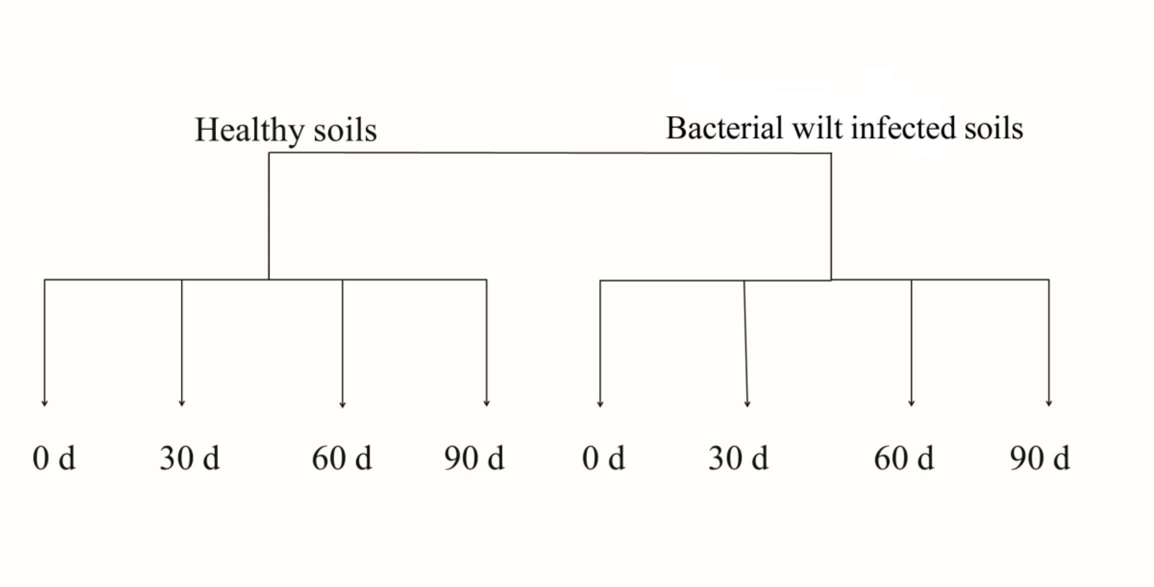

Supplement: Supplementary file 1 — Supplement Figures [file 41598_2017_472_MOESM1_ESM.pdf]
